# Editorial

**Published:** 1856-04

**Authors:** 


### Editorial.

### HYPERTROPHY OF THE GUMS.

The very interesting case of Hypertrophy, reported by Dr. Goddard of Louisville, will we are assured be of great interest to the profession. We should, however, regard the permanent cure of such a case, as very much depending on future constitutional treatment. To develop such a case there must be a marked constitutional diatheses favoring such a morbid growth. Shortly after the report of the case by Dr. Goddard, at our Society's meeting, we had an opportunity of seeing a somewhat similar case only however, in its incipient state. This occurred in the practice of Dr. C. Bonsall of this City, and was in the mouth of a young lady of some twenty years of age. It appeared to have originated from a slight injury to the inferior maxilla of the right side, and had commenced at the first bicuspid or cuspid, and not only these teeth were displaced by the growth, but also the second bicuspid and the front and lateral incisors. In this case there was some enlargement of the alveolar processes, yet it did not appear to extend below. Dr. Bonsall has since operated and removed the enlargment of the processes, the displaced teeth and also the gum, and at present the indications are a perfect cure. The Dr. has a plaster model of the case. Its history will likely be in the next number of the Register.

### ABSTRACTS.

We have not the room for our usual abstracts in this number. We shall likely occupy some space in the next, by reference to some valuable matters in the last numbers of our periodical. The April number of the News Letter is just received, and contains several interesting articles. This work we are glad to see is still enlarging its dimensions.

DENTAL GRADUATES

The Baltimore College gives the following list: A. H. Balderston, W. H. Hooper, and Thomas O. Walton, of Md., S. A. Bruce, and C. W. Reed of Va., C. M. King, G. W. Neidich, R. B. Reynolds, E. W. Swentzel, and E. Weiler, of Pa., T. J. Coopering, G. C. Lewis, and B. H. Padgett, of N. C., W. F. Eddington, of N. Y., R. C. Cyphers, of N. J., J. M. Lauck, D. C., J. W. Whitman of Miss.

The Philadelphia College gives the following list: J. Canning Allen, Philadelphia, Wm. T. Arrington, Tenn., James P. Brown, Va. Chas. H. Burr, Maine, Louis Martiny de Castro, Porto Rico, Antonie L. Coopat, Cuba, Francis Field, Mass., J. Foster Flagg, Philadelphia, James E. Garretson, Philadelphia, William Grimes, Ind., John W. Hunter, N. C., Jose G. Lopez, Porto Rico, Samuel Martin, M. D., N. C., A. F. McClain, La., Robert McClellon, Pa., Charles Niel, M. D., Philadelphia, Henry B. Parry, Lancaster, Pa., Allen W. Reid, Norristown, W. Bartling Robins, Philadelphia, R. Woodward Robinson, N. Y., John Z. Stanger, N. J., James K. Whiteside, Pa.

The Ohio College of Dental Surgery, graduated ten. The list will be found with the Proceedings of the Ohio Dental College Association in the present number of the Register.

---

HONORARY DEGREES.

We know not to what extent these have been granted this year. We can however, say for the Ohio College, that none were granted, and that those *most* entitled to such honors, were more anxious to maintain the honors of the profession and the school—caring more for these than the former. This however is always the case. We have known instances where such honors have b een *very* assiduouslysought, and after having been obtained, they appear to think that the Institution has been lowered by granting their request, and indeed such has been the case, but it is rather strange that such should so understand it themselves. There are, however, those who by long professional experience and high reputation, are fully entitled to such honors. Those who care more for professional excellence than those selfish feelings bound up in their own pecuniary affairs—those in fine who are willing to strive, ever, and ever for the advancement of Dental science.

We are glad to see that the Philadelphia College has taken a high stand on this subject, and that the Faculty prefer resignation in a body to being compelled to grant degrees where they do not think proper. We hope that the trustees will see the false position in which they are placed, and retrace their false steps and thus save an Intstitution so much needed.

We are well aware that the Faculty do not confer degrees, yet they are the ones always held responsible for all conferred, and should have a voice in the matter. Self respect should make any Faculty resign if this is denied them.

DR. GODDARD, OF LOUISVILLE, KY.

We cannot say but we regret to announce to the profession that this gentleman designs retiring from the arduous duties of the practice. We regret it on account of the high stand the Doctor maintains professionally and also on account of the many warm friends he has among his patients. But the fact is dental practice is laborious, and does not pay half as well as many other pursuits in life, and hence, with us, so many who have spent the prime of their life in the struggle for fame and excellence in the profession, get discouraged and tired just when they should leisurely recline on their laurels and reap a rich harvest. We cannot but regard it as right and proper after years of toil and labor in which the professional man has been perfecting himself in all that pertains to the duties devolving on him, he should be so much better paid for what he does, as that he may labor less and make more. The Dr. has to our knowledge a fine practice, and could without much effort give the same to a competent person.

OFFICE AND PRACTICE FOR SALE.

We are requested by Dr. G. S. Beatty, of Canton, Ohio, to say that he will sell out and leave his fine location and practice, to a good competent operator—one that he can recommend to his patrons.

Canton is one of the most desirable and flourishing interior towns in the State, and Dr. Beatty has had almost the entire practice of the place for ten or fifteen years—population near 8000.

DENTAL ASSOCIATION.

We have a circular from the following members of the profession at St. Louis, inviting the profession to meet with them and unite in forming an Association for the advancement of Dental science. We should like to have been with them, but could not at the time. The names attached to the circular should insure success, and hence we hope soon to hear of an association formed on good foundations.

The circular has the following names attached: C. W. Spalding, Edward Hale, Isaiah Forbes, S. Dunham, Henry Barron, G. H. Perine, C. W. Forbes, G. G. Samuel, H. J. B. McKellops, Samuel A. Bartleson, Edward Hale, Jr., Aaron Blake

*Treatment of Displacements of the Uterus, with the Abdominal Spring Pessary* By J. McF. Gaston, M. D.—This is a small pamphlet, illustrating by plates, and describing the application of the instrument to the diseases above specified. It merits the notice of those concerned.

Published by Walker & Evans, Charleston, South Carolina.

*Prize Essay.*—The Prize Essay of the Mississippi Valley Association, revised and corrected, is now stereotyped, and will be issued immediately. The object of the association, in offering the prize, was to obtain a concise work, adapted to all classes of readers, filled with information calculated to impress on them the importance of early attention to the teeth, and correct dental operations. That the circulation of such a work would do much to suppress quackery, greatly advance the interests of the honest and honorable of the profession, and, at the same time, do much good to society in general, needs no demonstration. Two thousand copies were subscribed for, by members of the profession, at an evening meeting of the association. In its present form it is a neat pocket manual of seventy-two pages.

The subscription price to the profession and dealers, is,

In paper, per hundred,......................................$ 6 00

" flexible cloth, " ...................................... 15 00

Orders may be addressed to the publishing committee, which consists of J. Taft, James Taylor, and J. P. Ulrey. Those who desire to be supplied from the first issue should forward their orders immediately.

---

*An Essay on Intermittent and Remittent Fevers; with their Pathological Relation to Ozone.* By E. S. Gaillard, M. D.—The above is an Essay of fifty-nine pages. It was originally presented as an inaugural dissertation to the faculty of the Medical College of the State of South Carolina, and received the annual premium of said institution. Every part of it gives evidence of close thought and extensive research. To the Medical Profession, especially in the West and South, it must prove highly interesting; and the dental practitioner would not only learn much of the pathology of fevers by its perusal but also something of that curious agent "Ozone." It is published by Walker & Evans, Charleston, South Carolina.

---

### ALUMINUM.

This new and beautiful metal has recently been worked into foil for filling teeth, by Messrs. C. Abby & Sons.

We are indebted to these gentlemen for a specimen of this foil—which looks very well—and show the zeal with which these gentlemen aim to meet the wants of our profession. The metal possesses some good properties for plugging material—it is sufficiently indestructible. The color is good, and not oxidyzable in the mouth. There is however as yet a "sort of stiffness" about it which renders it not very manageable for filling teeth.

Messrs. Abby & Sons remark, that the "seams or fractures do not unite together under the hammer as gold. This is observable in the specimen sent us.

We think that it may however be ultimately used much in Dental practice, likely for plates. We are under many obligations for the specimen of foil sent us.

